# The Networked Data Lab: lessons from four years of analytical collaboration on common problems in health on social care

**DOI:** 10.23889/ijpds.v9i5.2788

**Published:** 2024-09-18

**Authors:** Hannah Knight, Caroline Fraser, Tom Prendergast, Chamut Kifetew

**Affiliations:** 1 The Health Foundation

## Objective

The Networked Data Lab (NDL) is a UK-wide network of analytical teams embedded in the health and care system. Each year, we support five local partners to acquire and link new health and social care datasets on a different topic. The NDL provides insights from this linked data to help UK policymakers improve health and care, leveraging public, patient, and stakeholder engagement to prioritise topics of shared local interest.

## Approach

Now in its fourth year, the NDL has commissioned a Learning Study (LS) to evaluate progress towards its aims. This employs a theory-based approach to test and assess key assumptions about programme impact through a Theory of Change. For the study, semi-structured interviews with analysts and stakeholders at the five NDL sites were conducted to identify factors influencing the programme’s impact.

## Results

Drawing on interim LS findings, we will describe the types of impact the programme has achieved, and some of the key barriers and enablers to analytical collaboration across geographic and organisational boundaries. Themes include senior sponsorship; stakeholder input into problem definition; information governance and tools and skills. Themes will be illustrated with case studies drawn from the different NDL topic areas, including children and young people’s mental health and unpaid carers.

## Conclusions and Implications

The Networked Data Lab shows that data linkages can provide unique insights that local service planners can act on, but highlights a complex set of conditions that need to be met to enable impactful analytics using real-world health and care data.

